# Time trend analysis of leisure-time activity participation among young-old adults in China 2002–2018

**DOI:** 10.1186/s12889-022-12838-1

**Published:** 2022-03-02

**Authors:** Joelle H. Fong, Qiushi Feng, Wei Zhang, Huashuai Chen

**Affiliations:** 1 Lee Kuan Yew School of Public Policy, National University of Singapore, 469C Bukit Timah Road, National University of Singapore, Singapore, Singapore; 2 Department of Sociology & Centre for Family and Population Research, National University of Singapore, Singapore, Singapore; 3 Department of Sociology, University of Hawaiʻi at Mānoa, Honolulu, USA; 4 School of Business, Xiangtan University, Hunan, China

**Keywords:** Leisure activity, Population aging, China, Temporal trends

## Abstract

**Background:**

To examine the time trends of leisure activity engagement among young-old adults aged 65–74 in China over a 16-year period.

**Methods:**

Data for a nationally representative sample of young-old adults was sourced from the 2002–2018 Chinese Longitudinal Healthy Longevity Survey (*N* = 9504). Generalized estimating equations regressions were implemented to assess temporal trends for 10 different leisure-time activities. We also evaluated time trends for solitary versus social leisure-time activities.

**Results:**

Young-old adults were less likely to engage in any form of social activities (e.g. participate in social events) over time, controlling for other confounders such as age, sex, education, income, and health characteristics. Trends in outdoor activities participation and tourism also declined over 2002-2014, but reversed in 2018. In contrast, solitary leisure activities (e.g. watching TV) became more popular. There was a significant spike in the likelihood of keeping pets from 2011 onwards, especially among urbanites.

**Conclusions:**

The future elderly in China have tended towards home-bound and solitary leisure activities over time, which warrants policy attention and public health interventions to reverse such trends.

**Supplementary Information:**

The online version contains supplementary material available at 10.1186/s12889-022-12838-1.

## Background

Leisure activities usually constitute a large part of daily life for adults in early old age who are retired or transiting into retirement. These activities such as reading, exercising, and socializing with friends have often been considered to be beneficial to mental and physical health, and have been associated with higher quality of life, successful transition into retirement, better cognitive capacities, lower risk of chronic conditions, and reduced mortality risk [1–8]. Active participation in leisure-time physical and/or social activities are important to improving health and well-being among older populations. Thus, time trend analysis and surveillance studies on leisure activity engagement using longitudinal data are greatly valued in public health and medical sciences research. Numerous studies have documented positive temporal developments in Western countries. For instance, it is evidenced that the proportion of older Americans reporting *no* leisure-time physical activity engagement has steadily declined over 1990s-2010s [9]. Similar trends have been observed in Spain, Australia, and other Western nations [10, 11]. It is reported that the prevalence of leisure-time physical activity among Spanish elderly men aged 65 and above increased from 25.5% in 1987 to 69.6% in 2006, while that for elderly women also increased from 12.7 to 54.6% over the same period [10].

Fewer studies have examined leisure-time activity engagement among older populations in aging Asian societies such as China and Taiwan. Also, of concern, is that those which did have mostly documented worrying trends. Using a representative longitudinal sample of older Taiwanese aged 70 and above, one surveillance study found that participation in walking, yard/gardening, and exercise among older Taiwanese adults declined significantly from 1999 to 2007 [12]. Between 1998 and 2008, elderly residents in Shanghai China were more likely to engage in leisure-time habits that are not related to physical activity (e.g. watching TV), while the odds of them exercising regularly declined over time [13]. A time trend analysis of leisure activity engagement using a large database of Chinese oldest-old revealed that the adults aged 80 and above in China have become more sedentary and solitary from 1998 to 2018 [14].

This paper aims to investigate the trend of leisure-time activity participation among young-old adults aged 65-74 in China from 2002 to 2018. We conducted time trend analyses of the prevalence of 10 different leisure activities using a multivariate regression framework. In addition, following prior studies [5, 15, 16], we grouped the various activities into two aggregate clusters—solitary versus social—for broader analysis. This analytical strategy provides insights into how trends vary across individual activities, as well as between clusters, which may be policy implicative. Young-old adults represent the future elderly in China. Our focus on this demographic segment is also consistent with a large body of work in gerontology and sociology which has distinguished the young-old from the old-old [17–20]. Based on these studies, the older adult population can be divided into three life-stage subgroups: the young-old (approximately 65–74), the middle-old (ages 75–84), and the old-old (over age 85). Not only may young-old adults be wealthier, better educated, and more digitally savvy than previous generations, they also have potential to develop new leisure habits and increase community participation as they transit into retirement which allows for potential policy interventions.

Our 16-year investigation period of 2002-2018 covers a period of rapid modernization and urbanization in China. Since China acceded to the World Trade Organization in 2001, improvements in its socialist market economy system resulted in unprecedented economic growth. China’s GDP per capita increased by about four folds from about US$1770 in 2000 to US$7800 in 2018 [21]. Currently, about half of the nation’s population live in urban areas. The urbanization ratio is expected to reach almost 80% over the next few decades [21]. At the same time, China’s population is rapidly aging. Between 2000 and 2020, the proportion of adults 65 and above almost doubled from 6.8 to 12.0% [22]. By 2050, it is estimated that the country will host more than 350 million adults age 65 and above. Our study therefore also serves to inform how increasing affluence, modernization of lifestyle, and urbanization may have transformed Chinese adults’ leisure habits over time.

## Methods

### Study setting and participants

We drew data from the 2002-2018 Chinese Longitudinal Healthy Longevity Surveys (CLHLS). The CLHLS is a nationwide survey conducted in 631 randomly selected cities and counties in 22 provinces, covering about 85% of the total population of China. More than 8500 individuals aged 80 and above were interviewed at the 1998 baseline survey, and re-interviewed face-to-face every two to 3 years. With additional support from United Nations Population Fund (formerly the United Nations Fund for Population Activities), the China National Social Science Foundation, and other resources, the 2002 wave of the CLHLS survey expanded to cover younger adults below age 80. The CLHLS targets to interview all centenarians in the sampled counties and cities. Since 2002, for every two interviewed centenarians, approximately three younger adults aged 65-79 living nearby were interviewed. “Nearby” refers to persons living in the same village or street, or in the same or neighboring county or city. The inclusion of these younger respondents not only significantly expanded the scope of the survey, but also helped refreshed the sample.

The CLHLS also adopted a targeted random-sample design to ensure representativeness and individual-level weights are available. The survey was administered in the participants’ homes by well-trained interviewers from the local centres for disease prevention and control or university students. Overall response rate is about 90% each wave. Ethical approval for the CLHLS was obtained from the Research Ethics Committees of Peking University and Duke University, and all participants provided written informed consent. A complete description of the CLHLS is given elsewhere [23, 24]. We used data collected in waves 2002, 2005, 2008, 2011, 2014, and 2018 (total of 16 years). We pooled observations across all six waves and the final sample included 9504 subjects aged 65-74 with a total of 15,824 observations. Individuals age 75 and older were not included in this analysis. Participants without data on leisure activities were also excluded. Data for most covariates were complete except for education and income. Nonetheless, missings for these two covariates were generally < 5%and were imputed using regression imputation methodology. All methods were performed in accordance with the relevant ethical guidelines and regulations.

### Measurement of leisure activities

Self-reported information on engagement in various leisure activities was collected through CLHLS’ in-home interviews. Our study focused on 10 leisure activities, including reading newspapers/books, watching TV or listening to radio, doing housework, keeping pets/domestic animals, gardening, playing cards/mah-jong, attending social activities, joining outdoor activities, regular exercise, and tourism (i.e. travel at least once in the past 2 years). The participants reported their frequency of participation as almost every day, at least once a week, at least once a month, less than once a month, or never, for each of the activities except for regular exercise and tourism where the response categories are dichotomous (yes/no). Since our focus is on temporal trends rather than how intensively each leisure activity is performed, and for consistency, we coded all the leisure activities as dichotomous variables (where applicable, never is the “no” category and the remaining responses are combined into “yes”).

The ten leisure activities were classified as either ‘social’ or ‘solitary’ drawing on the various conceptualizations of leisure activities used in past studies. In particular, Sun and Liu [16] distinguished between solitary and social forms of leisure-time activities and proposed three different categories, namely solitary-active, solitary-sedentary, and social activities. Zhang and colleagues [5, 15] conceptualized three dimensions of leisure activities—cognitively-stimulating, socially engaged, and physically demanding—in examining the mediating effect of such activities on psychological distress. In this study, we adopted a simple classification of just two categories—‘solitary’ versus ‘social’—so as to contrast activities that are primarily conducted alone to those that are performed with others (group-based). As such, in this sense, our reference to ‘solitary’ is an activity that is usually performed alone and unaccompanied, which in the case of keeping pets say, include feeding and cleaning up after them. The five social activities comprised playing cards or mah-jong, attending social activities, participating in outdoor activities, regular exercise, and travelling at least once in the past 2 years, while the five solitary activities included reading newspapers or books, watching TV or listening to radio, doing housework, gardening, and keeping pets or domestic animals. “Regular exercise” was grouped under social leisure since many older Chinese adults prefer to exercise in groups; for instance, practicing Tai Chi together in community parks [25].

### Measurement of covariates

Several potential confounders included in our analyses were selected based on previous literature [8, 13, 14]. The sociodemographic covariates included age (in years), sex (male or female), place of residence (urban or rural), education (in years of schooling), and marital status (married and living with spouse or no). Social network and economic variables include household income (in logs), number of living children (in units), and co-residence with adult children (yes or no). To assess the participants’ functional status, the Katz activities of daily living (ADL) Scale was used which comprises bathing, dressing, toileting, transferring, eating, and continence [26]. Young-old adults who were unable to independently perform at least one of these tasks were defined as having an ADL limitation. Similarly, persons who were unable to independently perform at least one instrumental activity of daily living (IADL) item (go shopping, take public transportation, visit neighbours, cook a meal, wash clothing, walk 1 km, lift a heavy bag of groceries, as well as crouch and stand up three times) were defined as having an IADL limitation. Respondents reported their health status as very good, good, so-so, bad, and very bad. The responses were dichotomized into bad/very bad (poor self-rated health) or very good/good/so-so.

### Analytical strategy

We reported the prevalence rate of engagement in each type of leisure activity across all survey waves for comparative purposes. Generalized estimating equations (GEE) regression models were then implemented to evaluate the temporal trends in the prevalence of the ten individual leisure activities. GEE is a general statistical approach to fit a marginal model for longitudinal data analysis, and it has been popularly applied in biomedical and health studies [27, 28]. A separate set of GEE regression is performed for each individual leisure activity considered, where the dependent variables in each regression are the repeated measurements of leisure activity participation through time. A key strength of the GEE models is that they can address the correlation of repeated measures of the same individuals in the panel data over time (e.g., persons included in three or four cohorts in this study) through the exchangeable correlation structure. Since our focus was on temporal trends, the survey year was the key independent variable of interest (with year 2002 as the reference category). All regressions controlled for age, sex, residence, education, marital status, household income, number of living children, co-residence with adult children, ADLs, IADLs, and self-rated health.

Our main analysis focused on the 10 different types of leisure activities individually, as well as by cluster (social versus solitary). The odds ratios (ORs) and 95% confidence intervals from the GEE models for each activity analyzed are reported. To allow for cluster-level comparison, we synthesized part of the regression results into a graphical format. We also conducted sensitivity analyses in term of stratifying the sample by sex and place of residence. This is to account for possible disparity in leisure-time habits between males and females, and the rural-urban divide in terms of infrastructure, socioeconomic status, and other aspects in China. The ORs and accompanying 95% confidence intervals from these additional subgroup analyses are also reported. Individual-level age-sex-rural/urban-specific sample weights provided in the dataset were applied in all analyses to derive a representative population-based sample of young-old adults in China [24]. All statistical analyses were performed using STATA version 16.0 (StataCorp, College Station, TX, USA).

## Results

### Prevalence of leisure activity participation

Table 1 shows the weighted prevalence rates of young-old Chinese adults engaged in each leisure activity across the six survey waves. In the Table, the individual activities are ordered by their 2018 prevalence rates within the two clusters. Across the 16-year study period, watching TV was the top-ranked leisure activity among Chinese adults aged 65-74. Doing housework was more common than other solitary free-time activities such as keeping pets, reading newspapers or books, and gardening. The most prevalent social activities (irrespective of study year) were joining outdoor activities and regular exercise. By and large, participation in social leisure activities was less prevalent than solitary leisure activities among young-old adults in China. In 2018, the prevalence rates of the five social leisure activities ranged from 23.5-85.2%, while those of the five solitary leisure activities ranged from 28.4-90.5%.

**Table 1:** Descriptive statistics across survey waves

Year	2002	2005	2008	2011	2014	2018
Number of observations	3279	3313	2905	1923	1150	3254
*Solitary leisure activities*						
% Watching TV	86.0	89.6	91.3	90.3	93.9	90.5
% Doing housework	85.2	86.6	87.1	83.2	82.1	85.0
% Keeping pets/ domestic animals	47.1	44.2	39.2	40.3	46.3	40.5
% Reading newspapers/books	31.8	36.4	35.4	36.6	37.9	32.4
% Gardening	23.3	27.9	24.1	32.2	39.8	28.4
*Social leisure activities*						
% Joining outdoor activities	84.6	81.0	79.9	76.9	73.0	85.2
% Regularly exercise	38.4	42.0	42.1	42.8	31.0	41.2
% Playing cards/mah-jong	27.3	28.4	29.8	24.9	28.6	29.8
% Travel at least once in past 2 years	13.4	13.8	12.4	14.4	13.5	23.8
% Attending social activities	23.0	25.3	24.7	25.1	29.3	23.5
*Covariates*						
Mean age (sd)	69.0 (2.8)	69.1 (2.8)	69.2 (2.8)	69.3 (2.8)	69.8 (2.6)	68.8 (2.8)
% Female	50.5	50.0	50.0	47.4	48.2	50.7
% Rural residence	64.1	56.6	56.5	53.5	56.9	50.1
% Married & living with spouse	69.0	70.5	72.9	74.5	76.3	80.9
Mean years of education (sd)	3.0 (3.8)	3.6 (4.1)	4.0 (4.1)	4.3 (3.8)	4.4 (3.6)	5.3 (4.2)
Mean income, logged (sd)	8.3 (1.4)	8.6 (1.6)	9.2 (1.4)	9.4 (1.5)	9.7 (1.3)	10.0 (1.5)
Number of living children (sd)	4.0 (1.8)	3.7 (1.6)	3.5 (1.5)	3.2 (1.5)	3.0 (1.4)	1.4 (1.0)
% Co-reside with children	49.4	47.7	44.2	40.2	28.8	42.1
% Any ADL limitation	4.7	3.5	2.8	7.3	7.4	3.6
% Any IADL limitation	25.8	25.9	19.0	27.7	27.0	23.3
% Poor self-rated health	13.6	14.2	15.1	15.2	10.6	11.9

Notes: *sd* standard deviation

Statistics are shown for the weighted sample based on individual-level age-sex-rural/urban-specific sample weights; see text.

In each survey wave, approximately half of the respondents were female and mean age was 68.8-69.0 years. A large majority of the age 65-74 respondents were married and living with spouse, and about two in five also lived with their adult children. The weighted statistics reflected the economic progress and urbanization of China over the past two decades. Specifically, average years of education attained increased in each wave, as did average household income. However, family sizes (given by number of children) shrank. The percentage living in rural regions declined over the observation period (from 64.1% in 2002 to 50.1% in 2018) but nonetheless, remained substantial. Health and functional disability characteristics did not vary substantially across waves. Reduced sampling (owing to funding constraints) in waves 2011 and 2014 accounted for the smaller number of observations in those two waves. Attrition through mortality or loss to follow-up in the sample was not high since the respondents were only in their 60s and 70s.

### Temporal trends of leisure activity participation

**Fig. 1:**
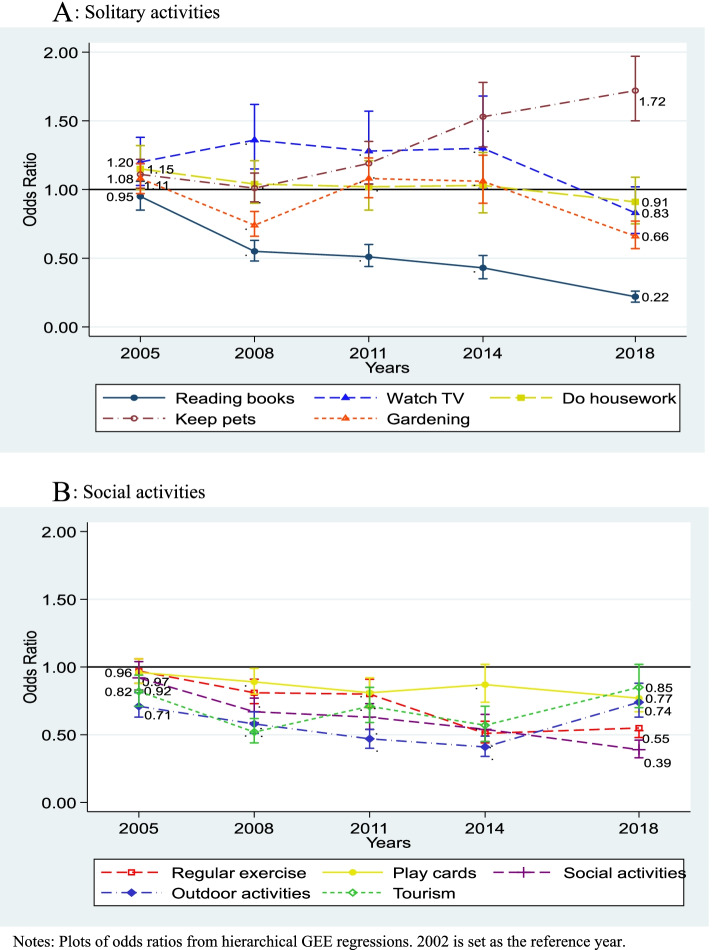
Temporal trends of leisure activity engagement among young-old adults in China, 2002–2018. **A **Solitary activities. **B **Social activities. Notes: Plots of odds ratios from hierarchical GEE regressions. 2002 is set as the reference year

To quantify temporal trends in the 10 leisure-time activities participation, we fitted 10 separate GEE regression models. The full sets of GEE regression results are available in Additional file 1: Appendix A. Given our focus is on studying trends, we extricated the estimated ORs of the relevant year dummies for each activity analyzed and plotted them in Fig. 1. The data points in the plots are the adjusted ORs from the GEE model; the whiskers are the corresponding 95% confidence intervals. Panel A illustrates the trends for the five solitary activities, while Panel B shows the trends for the five social activities. Thus, these graphs allow a visual comparison of temporal trends not only by type of activity, but also by cluster.

Focusing first on Panel A, we see that the population-based trends for three of the five solitary activities demonstrated relatively clear patterns. The odds of reading newspapers and books declined consistently throughout the 16-year period. However, as compared to 2002, young-old adults were also significantly more likely to watch TV during their free time from 2005 to 2014 (2005: OR = 1.20, CI = 1.03-1.38, *P* = 0.017; 2008: OR = 1.36, CI = 1.15-1.62, *P* < 0.001; 2011: OR = 1.28, CI = 1.04-1.57, *P* = 0.019; 2014: OR = 1.30, CI = 1.01-1.68, *P* = 0.042; see Panel A). There was also a clear pattern for likelihood of keeping pets, which trended upward substantially over 2011-2018. In contrast, the trends for doing housework and gardening were generally mixed.

Panel B of Fig. 1 displays the time trends for five social activities. The graph reveals that young-old adults in subsequent study years were less likely to engage in any form of social leisure activity as compared to 2002. The ORs for all five social activities were below one in each observation year (relative to base year), resulting in a strong overall downward trend for the social activity cluster. Among these activities, the odds of participating in social events (e.g. festival events, community celebrations), regular exercise, and outdoor activities decreased most prominently over the study period. For instance, as compared to 2002, young-old adults were significantly less likely to attend social activities during their free time from 2005 to 2018 (2005: OR = 0.92, CI = 0.83-1.04, *P* = 0.177; 2008: OR = 0.67, CI = 0.59-0.77, *P* < 0.001; 2011: OR = 0.63, CI = 0.54-0.73, *P* < 0.001; 2014: OR = 0.54, CI = 0.45-0.65, *P* < 0.001; 2018: OR = 0.39, CI = 0.33-0.46, *P* < 0.001; Panel B). The respondents were also significantly less likely to join in leisure outdoor activities over the same observation period (2005: OR = 0.71, CI = 0.63-0.81; 2008: OR = 0.58, CI = 0.51-0.67; 2011: OR = 0.47, CI = 0.40-0.54; 2014: OR = 0.41, CI = 0.34-0.49; 2018: OR = 0.74, CI = 0.63-0.88; *P* < 0.001 in all cases).

Comparing the two figures revealed an important finding. Our results showed that the overall time trend for the social activity cluster contrasted sharply with the overall trend for the solitary activity cluster. In the social activity cluster, the ORs for all the social activities were below one implying declined participation over time. Although the trends in tourism and joining outdoor activities showed signs of reversal post-2014, the likelihood of engaging in these activities were still lower than 2002. In the solitary activity cluster, the ORs for the solitary activities (except for reading newspapers or books) were mostly above one. Although the trends for doing housework, watching TV, and gardening, started to decline in the tail-end of the observation period, their associated ORs were predominantly above one implying increased participation over time.

A number of the control variables including sex and place of residence were also associated with leisure-time activities participation; see Additional file 1: Appendix A. As compared to males, females were more likely to do gardening and housework, as well as participate in outdoor activities, regular exercise, tourism, and social events. Young-old rural residents were less likely to watch TV, read, do gardening, and participate in all types of social activities, as compared to their urban counterparts. Educational attainment was positively associated with watching TV, reading, gardening, and all types of social activities. Being married was positively associated with keeping pets, reading, gardening, tourism, watching TV and attending social events. Having one or more ADLs or IADLs was negatively associated with all forms of leisure-time activities, regardless solitary or social.

### Sensitivity analysis

For sensitivity analysis, we stratified the sample by sex (male and female) and place of residence (urban and rural). The subgroups considered were “male, urban”, “male, rural”, “female, urban”, and “female, rural”. As with the main sample, we focus on the estimates relating to the relevant year dummies in order to analyze trends. The relevant estimated ORs and confidence intervals from the subgroup regressions of the solitary activities are presented in Table 2; detailed GEE regression results are given in Additional file 1: Appendix A. For the five solitary activities, we find that the increased likelihood of watching TV over time is observed across all subgroups and the slight trend reversal in 2018 appears to be attributable mainly to urban adults. This may be because young-old Chinese adults are now substituting TV with portable screen-time devices (e.g. iPads). Interestingly, subgroup differences in the odds ratio for keeping pets are quite pronounced. The ORs for urban males (females) increased from 1.41 (1.13) in 2005 to 4.28 (3.54) in 2018, whereas those for rural adults remained close to the 1.00 reference. This suggests that the upward trend in keeping pets was mainly driven by urbanites.

**Table 2:** Odds ratios from GEE Models: Temporal trends of solitary leisure activity engagement by sex and place of residence, 2002–2018

	Odds ratio (95% CI)				
	2005	2008	2011	2014	2018
*N*	*3313*	*2905*	*1923*	*1150*	*3254*
**Watching TV**					
* n (%)*	*2957 (89.6)*	*2667 (91.3)*	*1759 (90.3)*	*1059 (93.9)*	*2953 (90.5)*
All	1.20 (1.03,1.38)	1.36 (1.15,1.62)	1.28 (1.04,1.57)	1.30 (1.01,1.68)	0.83 (0.68,1.02)
Male, urban	0.93 (0.59,1.46)	1.25 (0.72,2.16)	1.41 (0.79,2.53)	1.34 (0.67,2.69)	0.54 (0.32,0.91)
Male, rural	1.29 (0.95,1.74)	1.32 (0.96,1.81)	1.44 (0.98,2.11)	1.12 (0.72,1.74)	1.08 (0.71,1.64)
Female, urban	1.21 (0.87,1.69)	1.35 (0.90,2.03)	1.15 (0.72,1.83)	0.95 (0.54,1.69)	0.62 (0.41,0.94)
Female, rural	1.24 (0.99,1.55)	1.45 (1.12,1.88)	1.19 (0.86,1.66)	1.70 (1.09,2.65)	1.03 (0.73,1.44)
**Doing housework**					
* n (%)*	*2848 (86.6)*	*2490 (87.1)*	*1593 (83.2)*	*948 (82.1)*	*2720 (85.0)*
All	1.15 (1.00,1.32)	1.04 (0.90,1.21)	1.02 (0.85,1.21)	1.03 (0.83,1.27)	0.91 (0.75,1.09)
Male, urban	1.29 (1.02,1.64)	1.32 (1.02,1.71)	1.28 (0.95,1.73)	1.45 (1.01,2.10)	1.09 (0.80,1.48)
Male, rural	1.25 (1.01,1.55)	1.06 (0.85,1.33)	0.91 (0.70,1.19)	0.99 (0.72,1.35)	0.90 (0.67,1.21)
Female, urban	0.75 (0.47,1.18)	0.79 (0.48,1.30)	1.04 (0.57,1.89)	0.79 (0.41,1.53)	0.56 (0.32,0.98)
Female, rural	0.93 (0.58,1.50)	0.80 (0.49,1.30)	0.82 (0.47,1.45)	0.90 (0.44,1.82)	1.06 (0.58,1.94)
**Keeping pets/domestic animals**					
* n (%)*	*1445 (44.2)*	*1154 (39.2)*	*747 (40.3)*	*494 (46.3)*	*1255 (40.5)*
All	1.11 (1.01,1.22)	1.01 (0.91,1.12)	1.19 (1.04,1.35)	1.53 (1.31,1.78)	1.72 (1.50,1.97)
Male, urban	1.41 (1.12,1.77)	1.55 (1.19,2.02)	2.44 (1.82,3.27)	3.28 (2.31,4.66)	4.28 (3.14,5.82)
Male, rural	1.08 (0.91,1.29)	0.93 (0.77,1.12)	0.92 (0.73,1.14)	1.19 (0.92,1.55)	1.06 (0.82,1.36)
Female, urban	1.13 (0.90,1.42)	1.15 (0.88,1.50)	1.87 (1.38,2.53)	2.48 (1.71,3.59)	3.54 (2.58,4.85)
Female, rural	1.02 (0.85,1.22)	0.82 (0.68,1.01)	0.69 (0.54,0.89)	0.95 (0.71,1.29)	0.81 (0.62,1.05)
**Reading newspapers/books**					
*n (%)*	*1214 (36.4)*	*978 (35.4)*	*679 (36.6)*	*406 (37.9)*	*1112 (32.4)*
All	0.95 (0.85,1.06)	0.55 (0.48,0.63)	0.51 (0.44,0.60)	0.43 (0.35,0.52)	0.22 (0.18,0.26)
Male, urban	0.97 (0.78,1.22)	0.44 (0.34,0.57)	0.42 (0.32,0.56)	0.36 (0.25,0.51)	0.16 (0.12,0.21)
Male, rural	0.77 (0.64,0.93)	0.55 (0.45,0.69)	0.58 (0.45,0.75)	0.48 (0.36,0.65)	0.32 (0.24,0.43)
Female, urban	1.18 (0.94,1.49)	0.55 (0.41,0.73)	0.49 (0.35,0.69)	0.37 (0.24,0.58)	0.21 (0.14,0.30)
Female, rural	1.20 (0.84,1.70)	0.98 (0.65,1.47)	0.63 (0.38,1.05)	0.88 (0.49,1.58)	0.24 (0.14,0.42)
**Gardening**					
*n (%)*	*926 (27.9)*	*685 (24.1)*	*608 (32.2)*	*377 (39.8)*	*959 (28.4)*
All	1.08 (0.97,1.19)	0.74 (0.66,0.84)	1.08 (0.94,1.23)	1.06 (0.90,1.25)	0.66 (0.57,0.77)
Male, urban	1.05 (0.86,1.27)	0.58 (0.46,0.73)	0.77 (0.60,1.00)	0.67 (0.48,0.93)	0.50 (0.38,0.65)
Male, rural	1.26 (1.00,1.58)	0.89 (0.69,1.14)	1.39 (1.05,1.84)	1.35 (0.98,1.85)	0.86 (0.62,1.19)
Female, urban	0.95 (0.77,1.18)	0.62 (0.49,0.80)	0.72 (0.55,0.94)	0.82 (0.59,1.14)	0.55 (0.42,0.73)
Female, rural	1.11 (0.87,1.42)	1.04 (0.79,1.37)	1.88 (1.38,2.55)	1.91 (1.32,2.78)	1.02 (0.73,1.43)

Note*. CI* confidence interval

The rows in italics show the number (and percentage) of persons in a given observation year who participated in that specific activity. The percentages are shown in parenthesis. Year 2002 is the reference year for comparison (that is, OR = 1.0). “All” refers to the full sample. In each cell, the adjusted odds ratio is reported and the corresponding 95% confidence interval is shown in parenthesis. The regressions for the full sample controlled for age, sex, education, place of residence, marital status, household income, number of living children, co-residence with adult children, any ADL limitation, any IADL limitation, and self-rated health. Individual-level weights from respective survey waves are used.

Table 3 turns attention to the five individual social activities. The GEE regression results for the full sample indicate downward trends in regular exercise, social events e.g. outdoor dancing, and outdoor activities participation. This observation of declined participation over time in exercise, social events, and outdoor activities participation is also generally consistent across various subgroups stratified by sex (male and female) and residence (urban and rural). Interestingly, we noted that the decline in regular exercise engagement over time was relatively more persistent among urban males than other subgroups. This may be because males are still traditionally considered the breadwinners in Chinese culture, and those living in urban areas are consistently pressured to strive for better economic opportunities. Additionally, the subgroup analysis by gender showed that the post-2014 reversal in declining tourism was mainly attributable to increased participation in travel and tourism among females rather than males. A possible reason is the growing financial independence among Chinese women, which form a growing majority of the nation’s domestic and outbound travel markets.

**Table 3:** Odds ratios from GEE Models: Temporal trends of social leisure activity engagement by sex and place of residence, 2002–2018

	Odds ratio (95% CI)				
	2005	2008	2011	2014	2018
*N*	*3313*	*2905*	*1923*	*1150*	*3254*
**Joining outdoor activities**					
*n (%)*	*2685 (81.0)*	*2307 (79.9)*	*1445 (76.9)*	*846 (73.0)*	*2760 (85.2)*
All	0.71 (0.63,0.81)	0.58 (0.51,0.67)	0.47 (0.40,0.54)	0.41 (0.34,0.49)	0.74 (0.63,0.88)
Male, urban	0.85 (0.62,1.16)	0.76 (0.54,1.07)	0.55 (0.38,0.79)	0.40 (0.27,0.60)	0.67 (0.47,0.96)
Male, rural	0.66 (0.52,0.83)	0.50 (0.39,0.63)	0.39 (0.30,0.51)	0.33 (0.24,0.45)	0.76 (0.55,1.04)
Female, urban	0.81 (0.60,1.09)	0.76 (0.54,1.05)	0.56 (0.39,0.79)	0.51 (0.33,0.78)	0.56 (0.39,0.80)
Female, rural	0.65 (0.52,0.81)	0.54 (0.42,0.69)	0.47 (0.35,0.62)	0.49 (0.35,0.70)	1.15 (0.83,1.61)
**Regularly exercise**					
*n (%)*	*1411 (42.0)*	*1190 (42.1)*	*813 (42.8)*	*388 (31.0)*	*1364 (41.2)*
All	0.97 (0.88,1.06)	0.81 (0.73,0.91)	0.80 (0.70,0.91)	0.51 (0.44,0.60)	0.55 (0.48,0.63)
Male, urban	0.91 (0.74,1.11)	0.77 (0.62,0.97)	0.60 (0.47,0.78)	0.31 (0.23,0.43)	0.35 (0.27,0.46)
Male, rural	0.94 (0.77,1.13)	0.82 (0.67,1.02)	0.70 (0.55,0.89)	0.58 (0.43,0.77)	0.84 (0.64,1.10)
Female, urban	1.14 (0.93,1.38)	0.85 (0.67,1.06)	0.84 (0.65,1.09)	0.64 (0.46,0.88)	0.48 (0.37,0.63)
Female, rural	0.92 (0.75,1.13)	0.76 (0.60,0.96)	1.28 (0.97,1.69)	0.61 (0.42,0.87)	0.78 (0.58,1.05)
**Playing cards/mah-jong**					
*n (%)*	*948 (28.4)*	*824 (29.8)*	*523 (24.9)*	*342 (28.6)*	*962 (29.8)*
All	0.96 (0.88,1.06)	0.89 (0.80,0.99)	0.81 (0.71,0.92)	0.87 (0.74,1.02)	0.77 (0.67,0.88)
Male, urban	0.91 (0.76,1.09)	0.85 (0.69,1.05)	0.70 (0.54,0.90)	0.64 (0.46,0.87)	0.78 (0.61,1.00)
Male, rural	0.96 (0.81,1.14)	0.78 (0.64,0.95)	0.67 (0.53,0.85)	0.92 (0.70,1.21)	0.80 (0.61,1.04)
Female, urban	0.96 (0.78,1.18)	0.89 (0.70,1.13)	0.97 (0.74,1.28)	0.77 (0.54,1.10)	0.73 (0.54,0.98)
Female, rural	1.00 (0.81,1.24)	0.98 (0.77,1.24)	0.85 (0.62,1.18)	1.02 (0.70,1.47)	0.70 (0.51,0.96)
**Tourism/ travel at least once in past 2 years**					
*n (%)*	*457 (13.8)*	*340 (12.4)*	*294 (14.4)*	*152 (13.5)*	*788 (23.8)*
All	0.82 (0.71,0.94)	0.52 (0.44,0.62)	0.71 (0.59,0.85)	0.57 (0.45,0.71)	0.85 (0.70,1.02)
Male, urban	0.73 (0.57,0.94)	0.38 (0.29,0.51)	0.58 (0.43,0.80)	0.26 (0.17,0.41)	0.61 (0.44,0.84)
Male, rural	0.79 (0.57,1.09)	0.45 (0.31,0.65)	0.48 (0.32,0.72)	0.62 (0.40,0.96)	0.61 (0.39,0.95)
Female, urban	0.84 (0.63,1.12)	0.66 (0.49,0.89)	0.89 (0.64,1.24)	0.75 (0.49,1.15)	1.03 (0.74,1.45)
Female, rural	0.93 (0.66,1.31)	0.66 (0.45,0.97)	1.08 (0.69,1.69)	0.87 (0.50,1.52)	1.18 (0.72,1.94)
**Attending social activities**					
*n (%)*	*840 (25.3)*	*682 (24.7)*	*456 (25.1)*	*261 (29.3)*	*777 (23.5)*
All	0.92 (0.83,1.04)	0.67 (0.59,0.77)	0.63 (0.54,0.73)	0.54 (0.45,0.65)	0.39 (0.33,0.46)
Male, urban	0.98 (0.80,1.20)	0.58 (0.46,0.73)	0.56 (0.42,0.74)	0.46 (0.33,0.66)	0.34 (0.26,0.45)
Male, rural	0.89 (0.71,1.12)	0.63 (0.49,0.82)	0.51 (0.37,0.68)	0.42 (0.29,0.60)	0.43 (0.31,0.61)
Female, urban	1.05 (0.83,1.32)	0.75 (0.57,0.97)	0.89 (0.67,1.19)	0.84 (0.59,1.20)	0.41 (0.30,0.57)
Female, rural	0.80 (0.62,1.05)	0.78 (0.58,1.04)	0.65 (0.44,0.96)	0.61 (0.39,0.96)	0.42 (0.28,0.64)

Note*. CI* confidence interval

The rows in italics show the number (and percentage) of persons in a given observation year who participated in that specific activity. The percentages are shown in parenthesis. Year 2002 is the reference year for comparison (that is, OR = 1.0). “All” refers to the full sample. In each cell, the adjusted odds ratio is reported and the corresponding 95% confidence interval is shown in parenthesis. The regressions for the full sample controlled for age, sex, education, place of residence, marital status, household income, number of living children, co-residence with adult children, any ADL limitation, any IADL limitation, and self-rated health. Individual-level weights from respective survey waves are used.

## Discussion

Using data from a nationally representative sample, we characterized the types and temporal trends of leisure-time activity engagement of adults aged 65-74 in China for the period 2002–2018. To the best of our knowledge, this is perhaps the first study on this topic. Overall, we found that social leisure activities tended to be less prevalent than solitary leisure activities, across all years, in this nationally representative sample. Young-old Chinese adults in more recent years were less likely to engage in any form of social leisure activity as compared to 2002, resulting in a significant and persistent downward trend for the social activity cluster. In contrast, we found a mixed but generally upward trend for the solitary activity cluster (with the exception of the reading activity).

Our study shows that most Chinese young-old adults favored home-bound leisure activities such as watching TV and doing housework (as high as about 90%), and only a small proportion (12-24%) travelled for leisure. In contrast, about half of European individuals aged 65 and above had travelled out of their country at least once in 2018 [29]. We found that trends in home-bound and solitary activities have become more salient among the young-old in recent years, with the odds of participating in social events, regular exercise, and outdoor activities decreasing prominently over time. Rural residence, male, fewer years of schooling, and having one or more ADLs or IADLs were positively associated with lack of participation in various types of social activities. This is particularly worrisome given that the sampled young-old adults were still in relatively good health, with only a small percentage (< 5%) having any ADL limitations. In other words, they were not unduly constrained in going out and making social interactions if they had wished to. Combined with the results from a recent study on the temporal trends of leisure-time activity engagement among the Chinese oldest-old [14], we arrive at the perturbing conclusion that older population in China, as a whole, has become more solitary in recent years.

The home-bounded tendency among older Chinese adults is highly relevant to the ongoing social transformation in China. Since the 1980s, China has been experiencing the rapid urbanization: the proportion of Chinese urban residents increased from about 19.4 to 26.4% from 1980 to 1990. This further increased to 35.9% in 2000, and then to 49.2% in 2010 [21]. Our investigation period captured the fastest segment of urbanization in China. The rapid urbanization was accompanied by housing privatization in urban areas, which promoted privacy but disconnected social ties within neighbourhoods [30, 31]. In rural areas, the migration of rural youth to large cities depleted villages and the left-behind older adults often have to take care of their grandchildren. As a result of these interrelated social changes, older Chinese adults (both urban and rural) have been broadly experiencing the decline of social participation in their later life. Accordingly, more can be done to encourage older adults to re-connect with their neighbors and participate in social activities, for instance by building walkable and cohesive neighborhoods [32].

On a more positive note, our results showed that the downward trend for participation in outdoor activities and tourism begun to reverse in the 2014-2018 interval. The declining trend in regular exercise also appeared have flattened out during the same interval. Given that our empirical estimations have controlled for various socio-demographic personal characteristics, it is plausible that broader system-level changes are driving such reversals in trends. This may include policy interventions, for example, the Chinese government’s ‘Health China 2030’ plan that was issued in 2016. The plan seeks to promote people’s health over a ten-year period through various channels such as providing activity recommendations on television programs, providing free health consultation in community health care centres, and promoting health awareness and education [33]. These efforts may have helped, directly or indirectly, generate greater participation in some social activities. Another noteworthy trend is that urban Chinese young-old adults have become more inclined to raise pets over time. Our stratified analysis showed that the likelihood of keeping pets in 2018 was about four times higher among urban young-old adults, compared to the base year 2002. The large magnitude of the odds ratios underscores the importance of this rising trend. This is a new phenomenon which has not been reported in literature thus far. One possible explanation is that more Chinese young-old adults are now living alone. For example, the proportion of solo-living among older adults in general has grown from 9.6% in 1990 to 12.5% in 2010 and is almost 20% in 2020 [34]. Persons in empty-nested households tend to experience high levels of loneliness [35, 36]. While recent studies suggest that pet ownership can compensate for the lack of human companionship [37], it is still unclear whether pet companionship can actually provide health benefits to older adults [38].

We found that the choice of indoor leisure activities has evolved in China over the last two decades. Although watching TV remained most dominant and trended upwards from 2005 to 2014, its popularity is starting to decline among the young-old in recent years. This contrasts with trends for the oldest-old Chinese adults, for whom the odds of watching TV have persistently increased by two to three times over the past two decades [14]. It is possible that the recent decreased likelihood of watching TV among Chinese young-old adults, especially those living in urban areas, is due to the increased availability of substitutes. Young-old adults who are born in the early 1950s have higher levels of education on average and are more digitally savvy than their parents. Thus, they may prefer multi-functional screen-time devices such as personal computers, smart phones, and iPads for entertainment, rather than a simple TV.

By examining how young-old Chinese adults spend their leisure time in the most recent two decades, this study makes a timely call for interventions and policies in China so as to encourage its older population to be more active and social, which may allow them to reap benefits in later life. Population aging has been considered a worrisome trend for many developing nations, including China, mainly due to concerns involving the shortage of working age population and the underdeveloped social security system. Findings from this study suggest the urgency of changing the lifestyle of Chinese young-old adults, which should be a more cost-effective way in dealing with challenges of population aging compared to costly investments in medical infrastructures. Encouraging young-old adults to participate in more social and physical leisure activities requires support from families and communities to provide opportunities, infrastructures, and facilities. Overall, our findings are consistent with a small handful of surveillance studies which have shown that older adults in aging Asian societies are becoming more sedentary over time [12–14]. With the increased availability of longitudinal datasets on aging going forward, more research can be conducted to monitor time-trends in leisure activity engagement among these older populations.

### Limitations and strengths

The present study is the largest prospective cohort study to investigate the temporal trends in leisure activities in young-old adults in China. The strengths of this study are its community-based, prospective design, relatively long observation duration, a nationally representative sample of the young-old, and the adjustment for potential confounders within the empirical framework. An additional strength of our study is our primary focus on young-old adults, who have enormous potential in developing new leisure-time activity habits and community participation as transit into their retirement phase. Even if retired, they are still likely in relatively good health and in the company of their spouses. Prospective cohort studies on leisure-time activities are valuable and useful in helping inform public health interventions and policies in developing societies. Nonetheless, these require panel or longitudinal data and sufficiently long follow-up periods. Our 16-year investigation period of 2002-2018 covers a reasonably long observation period, and also captures an important time frame defining modernization and urbanization in China. To the best of our knowledge, our study is the first to analyze time trends of leisure-time activity participation among young-old adults in China from 2002 to 2018**.**

This study has some limitations that future research can remedy. One limitation is that the activity measures were self-reported and might be subject to imprecise measurement. For instance, there may be recall biases given that the respondents were asked about events experienced in the previous 12 to 24 months. In our case, the fairly consistent responses elicited from each individual at different time-points for each activity provided some reassurance. Another potential limitation is that, despite carefully controlling for several established confounders, there is still a possibility of residual confounding by other unmeasured variables such as net wealth and employment status. Given that the data did not collate information on why respondents chose to participate in an activity or not, we were also unable to pinpoint the exact reasons for the time trends uncovered although some of the trends observed correspond closely to what is known generally about the Chinese society. Future research on leisure-time activities covering longer follow-up periods and with richer measures on the different activity types and reasons for participation will be required to investigate further the drivers of time trends in leisure activity engagement.

## Conclusion

Our study suggests that there is a need to strengthen the monitoring of leisure activity engagement across various segments of older adults, ranging from the young-old to the oldest-old, so that timely interventions can be taken to reverse negative trends that may have serious public health implications. The increased tendencies towards home-bound and solitary activities, as shown in this study, is particularly worrisome. As the population in China continues to age, public health professionals and policymakers may employ strategies to encourage greater leisure activity engagement across a more diverse and varied array of activities, which will ultimately yield better health outcomes and improved well-being in this population.

## Supplementary Information

**Additional file 1.**

## Data Availability

The data that support the findings of this study are available from the National Archive of Computerized Data on Aging at the Inter-University Consortium for Political and Social Research, University of Michigan, and can be made available to registered users. More information about the Chinese Longitudinal Healthy Longevity Survey can be found at (https://sites.duke.edu/centerforaging/programs/chinese-longitudinal-healthy-longevity-survey-clhls/).

## Abbreviations

ADL: Activities of daily living
CLHLS: Chinese Longitudinal Healthy Longevity Survey
GEE: Generalized estimating equations
IADL: Instrumental activities of daily living
OR: Odds ratio
TV: Television
