# Supplementary material for: Time trend analysis of leisure-time activity participation among young-old adults in China 2002–2018
Source: BMC Public Health. 2022 Mar 2;22:417. doi: 10.1186/s12889-022-12838-1 (PMC8889756; doi:10.1186/s12889-022-12838-1)
Supplement: Supplementary file 1 — Additional file 1. [file 12889_2022_12838_MOESM1_ESM.pdf]

Time trend analysis of leisure-time activity participation among young-old adults in China 2002–2018

<u>Supplementary material (online)</u>

Appendix A

Appendix Figure A1: Flow chart of the CLHLS study sample (2002-2018)

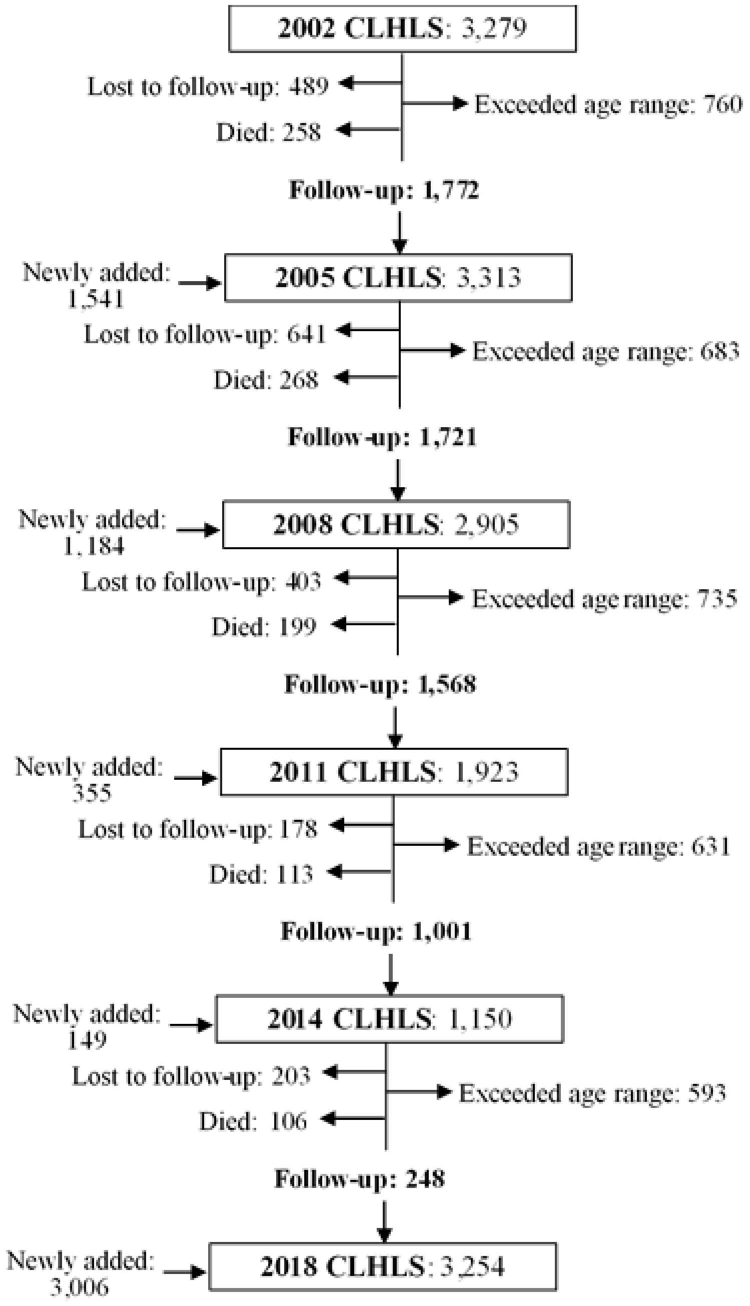

Appendix Table A1: Full GEE regressions for solitary leisure activities

	All<br>OR (95%CI)	Male, urban<br>OR (95%CI)	Male, rural<br>OR (95%CI)	Female, urban<br>OR (95%CI)	Female, rural<br>OR (95%CI)
**Dependent variable: Watching TV**					
Survey waves *(ref: 2002)*					
2005	1.20 (1.03, 1.38)**	0.93 (0.59, 1.46)	1.29 (0.95, 1.74)*	1.21 (0.87, 1.69)	1.24 (0.99, 1.55)*
2008	1.36 (1.15, 1.62)***	1.25 (0.72, 2.16)	1.32 (0.96, 1.81)*	1.35 (0.90, 2.03)	1.45 (1.12, 1.88)***
2011	1.28 (1.04, 1.57)**	1.41 (0.79, 2.53)	1.44 (0.98, 2.11)*	1.15 (0.72, 1.83)	1.19 (0.86, 1.66)
2014	1.30 (1.01, 1.68)**	1.34 (0.67, 2.69)	1.12 (0.72, 1.74)	0.95 (0.54, 1.69)	1.70 (1.09, 2.65)**
2018	0.83 (0.68, 1.02)*	0.54 (0.32, 0.91)**	1.08 (0.71, 1.64)	0.62 (0.41, 0.94)**	1.03 (0.73, 1.44)
Other controls:					
Age	0.96 (0.94, 0.98)***	0.98 (0.92, 1.04)	0.92 (0.89, 0.96)***	0.98 (0.93, 1.03)	0.97 (0.94, 1.00)**
Female	0.80 (0.71, 0.91)***	--	--	--	--
Rural residence	0.65 (0.58, 0.74)***	--	--	--	--
Years of schooling	1.14 (1.11, 1.16)***	1.13 (1.09, 1.18)***	1.10 (1.06, 1.15)***	1.19 (1.14, 1.25)***	1.14 (1.08, 1.19)***
Married	1.41 (1.24, 1.60)***	1.30 (0.87, 1.95)	1.56 (1.21, 2.01)***	1.35 (1.03, 1.76)**	1.41 (1.16, 1.73)***
Number of living children	1.02 (0.99, 1.06)	0.90 (0.81, 1.00)**	1.05 (0.98, 1.13)	1.01 (0.93, 1.10)	1.06 (1.00, 1.13)*
Co-residence with children	0.91 (0.80, 1.03)	1.14 (0.79, 1.62)	0.95 (0.74, 1.23)	0.94 (0.72, 1.23)	0.81 (0.66, 1.00)**
Log of income per capita	1.18 (1.14, 1.22)***	1.04 (0.92, 1.16)	1.18 (1.11, 1.25)***	1.31 (1.21, 1.42)***	1.16 (1.10, 1.22)***
1+ ADLs	0.39 (0.32, 0.49)***	0.35 (0.21, 0.56)***	0.33 (0.22, 0.50)***	0.35 (0.23, 0.53)***	0.59 (0.39, 0.88)***
1+ IADLs	0.66 (0.58, 0.74)***	0.51 (0.36, 0.74)***	0.76 (0.58, 1.00)**	0.65 (0.50, 0.85)***	0.64 (0.54, 0.77)***
Poor self-rated health	0.73 (0.63, 0.84)***	0.62 (0.42, 0.91)**	0.64 (0.48, 0.85)***	0.69 (0.50, 0.96)**	0.82 (0.66, 1.03)*
**Dependent variable: Doing house work**					
Survey waves *(ref: 2002)*					
2005	1.15 (1.00, 1.32)*	1.29 (1.02, 1.64)**	1.25 (1.01, 1.55)**	0.75 (0.47, 1.18)	0.93 (0.58, 1.50)
2008	1.04 (0.90, 1.21)	1.32 (1.02, 1.71)**	1.06 (0.85, 1.33)	0.79 (0.48, 1.30)	0.80 (0.49, 1.30)
2011	1.02 (0.85, 1.21)	1.28 (0.95, 1.73)*	0.91 (0.70, 1.19)	1.04 (0.57, 1.89)	0.82 (0.47, 1.45)
2014	1.03 (0.83, 1.27)	1.45 (1.01, 2.10)**	0.99 (0.72, 1.35)	0.79 (0.41, 1.53)	0.90 (0.44, 1.82)

	All OR (95%CI)	Male, urban OR (95%CI)	Male, rural OR (95%CI)	Female, urban OR (95%CI)	Female, rural OR (95%CI)
2018	0.91 (0.75, 1.09)	1.09 (0.80, 1.48)	0.90 (0.67, 1.21)	0.56 (0.32, 0.98)**	1.06 (0.58, 1.94)
Other controls:					
Age	0.96 (0.95, 0.98)***	0.98 (0.95, 1.01)	0.96 (0.94, 0.99)***	0.97 (0.92, 1.02)	0.91 (0.86, 0.97)***
Female	6.49 (5.73, 7.36)***	--	--	--	--
Rural residence	1.06 (0.95, 1.18)	--	--	--	--
Years of schooling	1.01 (0.99, 1.02)	1.02 (1.00, 1.04)*	1.00 (0.98, 1.03)	1.02 (0.98, 1.06)	0.92 (0.87, 0.98)**
Married	0.96 (0.85, 1.09)	0.95 (0.75, 1.21)	0.58 (0.47, 0.72)***	1.66 (1.22, 2.27)***	1.55 (1.11, 2.15)***
Number of living children	0.99 (0.96, 1.03)	0.95 (0.89, 1.01)	1.01 (0.96, 1.07)	1.01 (0.90, 1.12)	1.09 (0.99, 1.21)*
Co-residence with children	0.94 (0.84, 1.05)	0.94 (0.79, 1.13)	0.98 (0.83, 1.16)	1.07 (0.77, 1.50)	0.84 (0.58, 1.23)
Log of income per capita	1.01 (0.97, 1.05)	1.00 (0.93, 1.07)	1.06 (1.00, 1.12)*	0.91 (0.77, 1.07)	0.93 (0.80, 1.07)
1+ ADLs	0.16 (0.13, 0.19)***	0.17 (0.12, 0.25)***	0.30 (0.21, 0.43)***	0.10 (0.07, 0.14)***	0.12 (0.08, 0.18)***
1+ IADLs	0.38 (0.34, 0.43)***	0.38 (0.31, 0.47)***	0.40 (0.34, 0.48)***	0.30 (0.22, 0.41)***	0.34 (0.25, 0.48)***
Poor self-rated health	0.77 (0.67, 0.88)***	0.88 (0.69, 1.12)	0.83 (0.67, 1.01)*	0.68 (0.48, 0.96)**	0.55 (0.39, 0.77)***
**Dependent variable: Keeping domestic animals/pets**					
Survey waves *(ref: 2002)*					
2005	1.11 (1.01, 1.22)**	1.41 (1.12, 1.77)***	1.08 (0.91, 1.29)	1.13 (0.90, 1.42)	1.02 (0.85, 1.22)
2008	1.01 (0.91, 1.12)	1.55 (1.19, 2.02)***	0.93 (0.77, 1.12)	1.15 (0.88, 1.50)	0.82 (0.68, 1.01)*
2011	1.19 (1.04, 1.35)***	2.44 (1.82, 3.27)***	0.92 (0.73, 1.14)	1.87 (1.38, 2.53)***	0.69 (0.54, 0.89)***
2014	1.53 (1.31, 1.78)***	3.28 (2.31, 4.66)***	1.19 (0.92, 1.55)	2.48 (1.71, 3.59)***	0.95 (0.71, 1.29)
2018	1.72 (1.50, 1.97)***	4.28 (3.14, 5.82)***	1.06 (0.82, 1.36)	3.54 (2.58, 4.85)***	0.81 (0.62, 1.05)
Other controls:					
Age	0.96 (0.95, 0.97)***	0.95 (0.93, 0.98)***	0.98 (0.96, 1.00)*	0.97 (0.94, 1.00)*	0.95 (0.93, 0.98)***
Female	0.93 (0.86, 1.01)	--	--	--	--
Rural residence	2.62 (2.42, 2.83)***	--	--	--	--
Years of schooling	0.91 (0.90, 0.92)***	0.89 (0.87, 0.92)***	0.97 (0.95, 0.98)***	0.88 (0.85, 0.90)***	0.94 (0.91, 0.96)***
Married	1.38 (1.27, 1.51)***	1.41 (1.09, 1.81)***	1.50 (1.26, 1.77)***	1.32 (1.09, 1.59)***	1.47 (1.27, 1.71)***
Number of living children	1.10 (1.07, 1.13)***	1.20 (1.13, 1.28)***	1.05 (1.01, 1.10)**	1.13 (1.06, 1.20)***	1.04 (0.99, 1.09)*

	All OR (95%CI)	Male, urban OR (95%CI)	Male, rural OR (95%CI)	Female, urban OR (95%CI)	Female, rural OR (95%CI)
Co-residence with children	1.50 (1.38, 1.62)***	1.55 (1.29, 1.86)***	1.61 (1.39, 1.86)***	1.64 (1.36, 1.97)***	1.44 (1.23, 1.68)***
Log of income per capita	0.88 (0.85, 0.90)***	0.73 (0.67, 0.78)***	0.95 (0.91, 1.00)*	0.73 (0.67, 0.79)***	0.95 (0.91, 1.00)*
1+ ADLs	0.47 (0.39, 0.58)***	0.60 (0.36, 1.00)**	0.49 (0.35, 0.70)***	0.50 (0.32, 0.78)***	0.38 (0.26, 0.55)***
1+ IADLs	0.72 (0.66, 0.78)***	0.54 (0.42, 0.70)***	0.70 (0.59, 0.83)***	0.74 (0.61, 0.89)***	0.75 (0.65, 0.87)***
Poor self-rated health	0.79 (0.72, 0.88)***	0.90 (0.68, 1.20)	0.64 (0.53, 0.77)***	0.95 (0.75, 1.20)	0.82 (0.69, 0.97)**
**Dependent variable: Reading newspapers/books**					
Survey waves *(ref: 2002)*					
2005	0.95 (0.85, 1.06)	0.97 (0.78, 1.22)	0.77 (0.64, 0.93)***	1.18 (0.94, 1.49)	1.20 (0.84, 1.70)
2008	0.55 (0.48, 0.63)***	0.44 (0.34, 0.57)***	0.55 (0.45, 0.69)***	0.55 (0.41, 0.73)***	0.98 (0.65, 1.47)
2011	0.51 (0.44, 0.60)***	0.42 (0.32, 0.56)***	0.58 (0.45, 0.75)***	0.49 (0.35, 0.69)***	0.63 (0.38, 1.05)*
2014	0.43 (0.35, 0.52)***	0.36 (0.25, 0.51)***	0.48 (0.36, 0.65)***	0.37 (0.24, 0.58)***	0.88 (0.49, 1.58)
2018	0.22 (0.18, 0.26)***	0.16 (0.12, 0.21)***	0.32 (0.24, 0.43)***	0.21 (0.14, 0.30)***	0.24 (0.14, 0.42)***
Other controls:					
Age	1.00 (0.99, 1.02)	1.01 (0.98, 1.04)	0.99 (0.97, 1.02)	1.01 (0.98, 1.05)	1.01 (0.96, 1.06)
Female	0.46 (0.42, 0.51)***	--	--	--	--
Rural residence	0.52 (0.48, 0.57)***	--	--	--	--
Years of schooling	1.34 (1.32, 1.36)***	1.29 (1.25, 1.32)***	1.28 (1.25, 1.31)***	1.41 (1.37, 1.46)***	1.43 (1.36, 1.50)***
Married	1.19 (1.07, 1.33)***	1.39 (1.10, 1.75)***	1.34 (1.10, 1.64)***	1.03 (0.83, 1.26)	1.14 (0.85, 1.52)
Number of living children	0.93 (0.90, 0.96)***	0.91 (0.85, 0.96)***	1.02 (0.97, 1.07)	0.86 (0.80, 0.93)***	0.91 (0.84, 0.99)**
Co-residence with children	0.87 (0.79, 0.96)***	0.87 (0.73, 1.04)	0.93 (0.79, 1.10)	0.79 (0.64, 0.98)**	0.82 (0.60, 1.12)
Log of income per capita	1.27 (1.21, 1.33)***	1.29 (1.19, 1.40)***	1.23 (1.15, 1.32)***	1.28 (1.15, 1.42)***	1.31 (1.12, 1.53)***
1+ ADLs	0.57 (0.44, 0.74)***	0.42 (0.27, 0.65)***	0.73 (0.48, 1.11)	0.88 (0.56, 1.37)	0.37 (0.11, 1.23)
1+ IADLs	0.71 (0.63, 0.79)***	0.71 (0.57, 0.89)***	0.61 (0.50, 0.76)***	0.72 (0.59, 0.88)***	0.86 (0.64, 1.15)
Poor self-rated health	0.71 (0.62, 0.81)***	0.73 (0.57, 0.94)**	0.70 (0.56, 0.87)***	0.74 (0.56, 0.97)**	0.57 (0.38, 0.85)***
**Dependent variable: Gardening**					
Survey waves *(ref: 2002)*					
2005	1.08 (0.97, 1.19)	1.05 (0.86, 1.27)	1.26 (1.00, 1.58)*	0.95 (0.77, 1.18)	1.11 (0.87, 1.42)

	All OR (95%CI)	Male, urban OR (95%CI)	Male, rural OR (95%CI)	Female, urban OR (95%CI)	Female, rural OR (95%CI)
2008	0.74 (0.66, 0.84)***	0.58 (0.46, 0.73)***	0.89 (0.69, 1.14)	0.62 (0.49, 0.80)***	1.04 (0.79, 1.37)
2011	1.08 (0.94, 1.23)	0.77 (0.60, 1.00)*	1.39 (1.05, 1.84)**	0.72 (0.55, 0.94)**	1.88 (1.38, 2.55)***
2014	1.06 (0.90, 1.25)	0.67 (0.48, 0.93)**	1.35 (0.98, 1.85)*	0.82 (0.59, 1.14)	1.91 (1.32, 2.78)***
2018	0.66 (0.57, 0.77)***	0.50 (0.38, 0.65)***	0.86 (0.62, 1.19)	0.55 (0.42, 0.73)***	1.02 (0.73, 1.43)
Other controls:					
Age	1.02 (1.01, 1.03)***	1.02 (1.00, 1.05)	1.03 (1.00, 1.06)**	1.04 (1.01, 1.07)***	0.98 (0.95, 1.01)
Female	1.36 (1.24, 1.48)***	--	--	--	--
Rural residence	0.62 (0.57, 0.68)***	--	--	--	--
Years of schooling	1.10 (1.09, 1.12)***	1.09 (1.07, 1.11)***	1.09 (1.07, 1.12)***	1.12 (1.09, 1.14)***	1.12 (1.08, 1.15)***
Married	1.27 (1.15, 1.40)***	1.60 (1.26, 2.03)***	1.24 (1.00, 1.54)**	1.11 (0.94, 1.31)	1.33 (1.10, 1.61)***
Number of living children	0.96 (0.93, 0.98)***	0.95 (0.90, 1.01)*	0.95 (0.90, 1.01)*	0.97 (0.92, 1.03)	0.97 (0.92, 1.02)
Co-residence with children	0.90 (0.82, 0.98)**	0.97 (0.82, 1.13)	0.79 (0.66, 0.95)**	0.90 (0.76, 1.07)	0.89 (0.73, 1.08)
Log of income per capita	1.14 (1.10, 1.18)***	1.15 (1.08, 1.24)***	1.14 (1.06, 1.24)***	1.20 (1.12, 1.30)***	1.07 (0.99, 1.15)*
1+ ADLs	0.55 (0.43, 0.69)***	0.51 (0.32, 0.80)***	0.67 (0.41, 1.10)	0.53 (0.36, 0.77)***	0.75 (0.45, 1.23)
1+ IADLs	0.81 (0.73, 0.89)***	0.67 (0.54, 0.83)***	0.80 (0.64, 1.00)**	0.82 (0.69, 0.97)**	0.95 (0.79, 1.14)
Poor self-rated health	0.75 (0.67, 0.85)***	0.66 (0.51, 0.84)***	0.68 (0.52, 0.88)***	0.93 (0.74, 1.16)	0.76 (0.60, 0.97)**

Appendix Table A2: Full GEE regressions for social leisure activities

	All	Male, urban	Male, rural	Female, urban	Female, rural
	OR (95%CI)	OR (95%CI)	OR (95%CI)	OR (95%CI)	OR (95%CI)
**Dependent variable: Outdoor activities**					
Survey waves *(ref: 2002)*					
2005	0.71 (0.63, 0.81)***	0.85 (0.62, 1.16)	0.66 (0.52, 0.83)***	0.81 (0.60, 1.09)	0.65 (0.52, 0.81)***
2008	0.58 (0.51, 0.67)***	0.76 (0.54, 1.07)	0.50 (0.39, 0.63)***	0.76 (0.54, 1.05)*	0.54 (0.42, 0.69)***
2011	0.47 (0.40, 0.54)***	0.55 (0.38, 0.79)***	0.39 (0.30, 0.51)***	0.56 (0.39, 0.79)***	0.47 (0.35, 0.62)***
2014	0.41 (0.34, 0.49)***	0.40 (0.27, 0.60)***	0.33 (0.24, 0.45)***	0.51 (0.33, 0.78)***	0.49 (0.35, 0.70)***
2018	0.74 (0.63, 0.88)***	0.67 (0.47, 0.96)**	0.76 (0.55, 1.04)*	0.56 (0.39, 0.80)***	1.15 (0.83, 1.61)
Other controls:					
Age	1.02 (1.00, 1.04)**	1.02 (0.99, 1.06)	1.03 (1.00, 1.05)*	0.99 (0.95, 1.03)	1.02 (0.99, 1.05)
Female	1.15 (1.05, 1.26)***	--	--	--	--
Rural residence	0.71 (0.65, 0.78)***	--	--	--	--
Years of schooling	1.06 (1.04, 1.07)***	1.06 (1.03, 1.08)***	1.05 (1.03, 1.08)***	1.06 (1.03, 1.09)***	1.04 (1.01, 1.08)**
Married	0.98 (0.88, 1.08)	1.04 (0.79, 1.37)	0.88 (0.73, 1.08)	0.97 (0.79, 1.20)	0.96 (0.82, 1.14)
Number of living children	0.99 (0.96, 1.02)	1.01 (0.94, 1.08)	1.05 (1.00, 1.11)**	0.95 (0.89, 1.02)	0.96 (0.92, 1.01)
Co-residence with children	0.89 (0.81, 0.97)**	0.88 (0.72, 1.09)	0.88 (0.75, 1.04)	0.95 (0.77, 1.17)	0.82 (0.69, 0.99)**
Log of income per capita	1.06 (1.03, 1.10)***	1.08 (1.01, 1.17)**	1.06 (1.00, 1.11)**	1.13 (1.06, 1.21)***	1.03 (0.97, 1.09)
1+ ADLs	0.32 (0.27, 0.38)***	0.20 (0.14, 0.28)***	0.44 (0.31, 0.62)***	0.33 (0.23, 0.46)***	0.39 (0.27, 0.57)***
1+ IADLs	0.70 (0.64, 0.78)***	0.67 (0.52, 0.86)***	0.73 (0.60, 0.89)***	0.61 (0.49, 0.75)***	0.78 (0.66, 0.93)***
Poor self-rated health	0.75 (0.67, 0.84)***	0.73 (0.55, 0.96)**	0.72 (0.58, 0.88)***	0.67 (0.52, 0.85)***	0.84 (0.69, 1.03)*
**Dependent variable: Regular exercise**					
Survey waves *(ref: 2002)*					
2005	0.97 (0.88, 1.06)	0.91 (0.74, 1.11)	0.94 (0.77, 1.13)	1.14 (0.93, 1.38)	0.92 (0.75, 1.13)
2008	0.81 (0.73, 0.91)***	0.77 (0.62, 0.97)**	0.82 (0.67, 1.02)*	0.85 (0.67, 1.06)	0.76 (0.60, 0.96)**
2011	0.80 (0.70, 0.91)***	0.60 (0.47, 0.78)***	0.70 (0.55, 0.89)***	0.84 (0.65, 1.09)	1.28 (0.97, 1.69)*
2014	0.51 (0.44, 0.60)***	0.31 (0.23, 0.43)***	0.58 (0.43, 0.77)***	0.64 (0.46, 0.88)***	0.61 (0.42, 0.87)***

	All	Male, urban	Male, rural	Female, urban	Female, rural
	OR (95%CI)	OR (95%CI)	OR (95%CI)	OR (95%CI)	OR (95%CI)
2018	0.55 (0.48, 0.63)***	0.35 (0.27, 0.46)***	0.84 (0.64, 1.10)	0.48 (0.37, 0.63)***	0.78 (0.58, 1.05)
Other controls:					
Age	1.04 (1.03, 1.06)***	1.05 (1.02, 1.08)***	1.05 (1.02, 1.07)***	1.03 (1.00, 1.06)**	1.04 (1.01, 1.07)***
Female	1.12 (1.03, 1.21)***	--	--	--	--
Rural residence	0.44 (0.41, 0.47)***	--	--	--	--
Years of schooling	1.10 (1.09, 1.11)***	1.10 (1.08, 1.12)***	1.10 (1.08, 1.12)***	1.08 (1.06, 1.11)***	1.11 (1.07, 1.14)***
Married	0.91 (0.84, 1.00)**	1.04 (0.85, 1.28)	0.82 (0.68, 0.98)**	0.95 (0.81, 1.11)	0.83 (0.70, 0.98)**
Number of living children	0.94 (0.92, 0.97)***	0.94 (0.89, 0.99)**	0.99 (0.95, 1.04)	0.90 (0.85, 0.95)***	0.94 (0.90, 0.99)**
Co-residence with children	0.95 (0.88, 1.03)	1.04 (0.89, 1.21)	0.88 (0.75, 1.02)*	0.95 (0.81, 1.11)	0.92 (0.77, 1.10)
Log of income per capita	1.12 (1.08, 1.15)***	1.16 (1.09, 1.24)***	1.09 (1.02, 1.15)***	1.16 (1.08, 1.24)***	1.07 (1.00, 1.13)**
1+ ADLs	0.51 (0.42, 0.63)***	0.42 (0.29, 0.62)***	0.55 (0.36, 0.82)***	0.52 (0.36, 0.73)***	0.71 (0.46, 1.10)
1+ IADLs	0.81 (0.74, 0.89)***	0.76 (0.63, 0.93)***	1.09 (0.91, 1.31)	0.70 (0.60, 0.82)***	0.81 (0.68, 0.96)**
Poor self-rated health	0.77 (0.69, 0.86)***	0.75 (0.59, 0.94)**	0.73 (0.59, 0.90)***	0.69 (0.56, 0.86)***	0.91 (0.74, 1.12)
**Dependent variable: Playing cards/mah-jongg**					
Survey waves *(ref: 2002)*					
2005	0.96 (0.88, 1.06)	0.91 (0.76, 1.09)	0.96 (0.81, 1.14)	0.96 (0.78, 1.18)	1.00 (0.81, 1.24)
2008	0.89 (0.80, 0.99)**	0.85 (0.69, 1.05)	0.78 (0.64, 0.95)**	0.89 (0.70, 1.13)	0.98 (0.77, 1.24)
2011	0.81 (0.71, 0.92)***	0.70 (0.54, 0.90)***	0.67 (0.53, 0.85)***	0.97 (0.74, 1.28)	0.85 (0.62, 1.18)
2014	0.87 (0.74, 1.02)*	0.64 (0.46, 0.87)***	0.92 (0.70, 1.21)	0.77 (0.54, 1.10)	1.02 (0.70, 1.47)
2018	0.77 (0.67, 0.88)***	0.78 (0.61, 1.00)**	0.80 (0.61, 1.04)*	0.73 (0.54, 0.98)**	0.70 (0.51, 0.96)**
Other controls:					
Age	1.00 (0.98, 1.01)	1.00 (0.97, 1.02)	1.01 (0.99, 1.04)	1.00 (0.97, 1.02)	0.98 (0.95, 1.01)
Female	0.69 (0.63, 0.76)***	--	--	--	--
Rural residence	0.90 (0.83, 0.97)***	--	--	--	--
Years of schooling	1.04 (1.03, 1.05)***	1.02 (1.00, 1.03)*	1.04 (1.02, 1.07)***	1.07 (1.05, 1.09)***	1.11 (1.07, 1.15)***
Married	0.99 (0.90, 1.09)	0.83 (0.67, 1.02)*	1.15 (0.95, 1.39)	0.87 (0.72, 1.04)	1.01 (0.83, 1.23)
Number of living children	0.96 (0.94, 0.99)***	0.99 (0.94, 1.05)	0.98 (0.93, 1.03)	0.98 (0.92, 1.04)	0.92 (0.87, 0.98)***

	All	Male, urban	Male, rural	Female, urban	Female, rural
	OR (95%CI)	OR (95%CI)	OR (95%CI)	OR (95%CI)	OR (95%CI)
Co-residence with children	0.86 (0.79, 0.93)***	0.90 (0.77, 1.05)	0.80 (0.69, 0.94)***	0.87 (0.73, 1.04)	0.81 (0.66, 0.98)**
Log of income per capita	1.07 (1.04, 1.11)***	1.02 (0.96, 1.08)	1.14 (1.07, 1.20)***	1.05 (0.97, 1.13)	1.08 (1.01, 1.16)**
1+ ADLs	0.43 (0.34, 0.55)***	0.37 (0.23, 0.61)***	0.34 (0.21, 0.56)***	0.48 (0.31, 0.75)***	0.64 (0.38, 1.07)*
1+ IADLs	0.76 (0.70, 0.83)***	0.72 (0.60, 0.88)***	0.71 (0.59, 0.86)***	0.84 (0.70, 1.01)*	0.75 (0.62, 0.90)***
Poor self-rated health	0.81 (0.73, 0.90)***	0.94 (0.74, 1.18)	0.84 (0.68, 1.02)*	0.82 (0.65, 1.03)*	0.62 (0.48, 0.80)***
**Dependent variable: Tourism**					
Survey waves *(ref: 2002)*					
2005	0.82 (0.71, 0.94)***	0.73 (0.57, 0.94)**	0.79 (0.57, 1.09)	0.84 (0.63, 1.12)	0.93 (0.66, 1.31)
2008	0.52 (0.44, 0.62)***	0.38 (0.29, 0.51)***	0.45 (0.31, 0.65)***	0.66 (0.49, 0.89)***	0.66 (0.45, 0.97)**
2011	0.71 (0.59, 0.85)***	0.58 (0.43, 0.80)***	0.48 (0.32, 0.72)***	0.89 (0.64, 1.24)	1.08 (0.69, 1.69)
2014	0.57 (0.45, 0.71)***	0.26 (0.17, 0.41)***	0.62 (0.40, 0.96)**	0.75 (0.49, 1.15)	0.87 (0.50, 1.52)
2018	0.85 (0.70, 1.02)*	0.61 (0.44, 0.84)***	0.61 (0.39, 0.95)**	1.03 (0.74, 1.45)	1.18 (0.72, 1.94)
Other controls:					
Age	0.99 (0.97, 1.00)	0.99 (0.96, 1.02)	0.99 (0.96, 1.03)	0.96 (0.93, 0.99)**	1.00 (0.96, 1.05)
Female	1.45 (1.30, 1.62)***	--	--	--	--
Rural residence	0.56 (0.51, 0.62)***	--	--	--	--
Years of schooling	1.15 (1.13, 1.16)***	1.14 (1.11, 1.16)***	1.13 (1.10, 1.17)***	1.15 (1.12, 1.17)***	1.13 (1.09, 1.18)***
Married	1.13 (1.00, 1.28)*	1.44 (1.08, 1.93)**	1.28 (0.93, 1.76)	1.05 (0.86, 1.27)	1.00 (0.77, 1.30)
Number of living children	0.98 (0.94, 1.01)	0.97 (0.90, 1.04)	1.04 (0.96, 1.12)	0.95 (0.89, 1.02)	0.96 (0.89, 1.04)
Co-residence with children	0.84 (0.75, 0.94)***	0.59 (0.49, 0.72)***	0.83 (0.64, 1.08)	1.05 (0.85, 1.29)	1.01 (0.76, 1.33)
Log of income per capita	1.31 (1.23, 1.39)***	1.63 (1.45, 1.82)***	1.41 (1.25, 1.60)***	1.21 (1.08, 1.35)***	1.08 (0.96, 1.22)
1+ ADLs	0.59 (0.43, 0.81)***	0.54 (0.31, 0.95)**	0.82 (0.39, 1.76)	0.53 (0.31, 0.91)**	0.72 (0.34, 1.53)
1+ IADLs	0.67 (0.59, 0.77)***	0.61 (0.47, 0.81)***	0.68 (0.47, 0.96)**	0.72 (0.58, 0.89)***	0.62 (0.46, 0.82)***
Poor self-rated health	0.63 (0.54, 0.75)***	0.60 (0.43, 0.83)***	0.47 (0.29, 0.75)***	0.69 (0.52, 0.91)***	0.70 (0.49, 1.00)*
**Dependent variable: Attending social activities**					
Survey waves *(ref: 2002)*					
2005	0.92 (0.83, 1.04)	0.98 (0.80, 1.20)	0.89 (0.71, 1.12)	1.05 (0.83, 1.32)	0.80 (0.62, 1.05)

	**All**	**Male, urban**	**Male, rural**	**Female, urban**	**Female, rural**
	OR (95%CI)	OR (95%CI)	OR (95%CI)	OR (95%CI)	OR (95%CI)
2008	0.67 (0.59, 0.77)***	0.58 (0.46, 0.73)***	0.63 (0.49, 0.82)***	0.75 (0.57, 0.97)**	0.78 (0.58, 1.04)*
2011	0.63 (0.54, 0.73)***	0.56 (0.42, 0.74)***	0.51 (0.37, 0.68)***	0.89 (0.67, 1.19)	0.65 (0.44, 0.96)**
2014	0.54 (0.45, 0.65)***	0.46 (0.33, 0.66)***	0.42 (0.29, 0.60)***	0.84 (0.59, 1.20)	0.61 (0.39, 0.96)**
2018	0.39 (0.33, 0.46)***	0.34 (0.26, 0.45)***	0.43 (0.31, 0.61)***	0.41 (0.30, 0.57)***	0.42 (0.28, 0.64)***
Other controls:					
Age	1.00 (0.99, 1.02)	1.01 (0.98, 1.03)	1.02 (0.99, 1.05)	1.00 (0.98, 1.03)	0.97 (0.93, 1.00)*
Female	1.08 (0.99, 1.19)*	--	--	--	--
Rural residence	0.61 (0.56, 0.66)***	--	--	--	--
Years of schooling	1.11 (1.09, 1.12)***	1.10 (1.08, 1.12)***	1.07 (1.05, 1.10)***	1.13 (1.10, 1.15)***	1.11 (1.07, 1.15)***
Married	1.13 (1.02, 1.25)**	1.16 (0.93, 1.45)	1.48 (1.17, 1.88)***	0.98 (0.83, 1.17)	1.04 (0.84, 1.29)
Number of living children	0.93 (0.90, 0.96)***	0.91 (0.86, 0.97)***	0.96 (0.91, 1.01)	0.89 (0.84, 0.95)***	0.95 (0.90, 1.01)
Co-residence with children	0.81 (0.74, 0.89)***	0.79 (0.67, 0.94)***	0.88 (0.73, 1.06)	0.76 (0.64, 0.92)***	0.79 (0.62, 1.00)**
Log of income per capita	1.27 (1.21, 1.33)***	1.30 (1.19, 1.42)***	1.30 (1.18, 1.42)***	1.29 (1.18, 1.41)***	1.18 (1.06, 1.32)***
1+ ADLs	0.58 (0.45, 0.74)***	0.45 (0.27, 0.73)***	0.80 (0.47, 1.36)	0.70 (0.47, 1.06)*	0.53 (0.27, 1.02)*
1+ IADLs	0.74 (0.66, 0.82)***	0.66 (0.53, 0.84)***	0.65 (0.51, 0.83)***	0.71 (0.60, 0.86)***	0.95 (0.76, 1.19)
Poor self-rated health	0.77 (0.67, 0.88)***	0.76 (0.58, 1.00)**	0.65 (0.49, 0.85)***	0.77 (0.61, 0.98)**	0.91 (0.69, 1.20)
